# Hypoplastic left heart syndrome [HLHS]: treatment options in present era

**DOI:** 10.1007/s12055-018-0742-z

**Published:** 2018-10-31

**Authors:** Vivek Rai, Marcin Gładki, Mirosława Dudyńska, Janusz Skalski

**Affiliations:** Department of Pediatric Cardiac Surgery, Jagiellonian University Children’s Hospital, Ul. Wielicka 265, 30-663 Krakow, Poland

**Keywords:** Congenital heart defect, Single ventricle, Hypoplastic left heart syndrome

## Abstract

Hypoplastic left heart syndrome (HLHS) is the most severe form of congenital heart defect (CHD). The first successful intervention for it was undertaken by Norwood in 1983. Since then, there have been much development in the pre, intra, and postoperative treatment option in staged palliative surgical procedures. Early diagnostic management, prenatal interventions, innovative diagnostic methods, constantly modified surgical techniques, and hybridization contribute to a significant progress in treatment options. This will allow for defining an optimal strategy of improving survival and quality of life in HLHS patients. The development of intervention cardiology makes possible the stepwise treatment of the defect with one operation only. The first and third stage may be done by hybrid or interventional methods, then only the second stage of treatment needs to be done surgically. The world experience and all the available literature says that the 1st-stage procedure could be done now safely either directly or with a bridge to Norwood followed by the stage 2 with a Glen or Hemi-Fontan and followed by a Fontan down the lane surgically.

## Introduction

HLHS is the most severe form of CHD, representing cardiac malformations that are characterized by severe underdevelopment of the structures in the left heart-aorta complex, including the left ventricular cavity and mass. The term potentially denotes any malformation that involves underdevelopment of the left-sided cardiac structures from aortic stenosis and coarctation of the aorta to the other extremes involving aortic atresia, mitral atresia, and hypoplasia of the ascending aorta. Statistically, about 1.25% children with congenital heart defects are patients with single ventricle heart [1]. The majority of untreated newborns die within the first 7–10 days of life.

## Prenatal diagnosis

Development in the field of ultrasonography has allowed for recognizing early HLHS in the first trimester of fetal life, which helps in further planning and making decision whether to proceed with pregnancy. Anatomical defects in HLHS which worsen the prognosis are restrictive atrial connection, tricuspid regurgitation, and ventricular dysfunction [2]. The percentage of neurological complications is lower, due to early recognizing HLHS [3]. In some countries, where pregnancy termination rates are relatively high, a higher number of prenatal diagnoses caused a decrease in the birth rate of children with HLHS [4]. Fetal interventions in HLHS have been developed in order to improve hemodynamics and prevent the development of defects. Currently, two types of treatment are used in case of this defect: balloon septostomy in case of restrictions of the atrial septum and balloon valve surgery in cases of critical aortic stenosis. In both cases, the treatment is performed under guidance of ultrasound (fetal echocardiography). The procedure must be done in very experienced centers because in 20% of such interventions, loss of the fetus is involved [5].

During prenatal intervention, it is also possible to perform a thorough genetic diagnostic management. Indeed, the cause of the defect is multifactorial and not stable. The established risk of recurrence of the defect in future pregnancies is 2–4%, and in families with two children with this disadvantage, it reaches 25% [6].

## Heart transplant

Heart transplant in a newborn with HLHS is also an available treatment option. The procedure always entails the risk of organ rejection and complications associated with immunosuppression [infection, tumor]. In the present era, this method is still limited due to scarcity of donors [7]. In newborns, hearts to be transplanted may be harvested from non-compliant donors (ABO-incompatible), thanks to the immature immune system in neonates [8]. The quality of life after heart transplant is better as compared to patients with staged palliative surgery [9].

Heart transplantation is also a possible treatment modality in cases of extreme heart failure after the Fontan procedure, especially when the background is dysfunction of the ventricle. This is a high-risk procedure in patients being treated by heart transplant, but if successful, it provides a good quality of life in this group of patients [10]. Ventricular assist device (VAD) support can be used as a bridge to transplant in HLHS patients.

## Palliative treatment

Palliative treatment is the most commonly used method of treatment. This method includes three stages of treatment. The first stage called the Norwood operation takes place in the neonatal period, the second stage is the hemi-Fontan (HF) or Glenn procedures performed at around 4–6 months of life, and the final stage is the Fontan operation which takes place around 18–24 months of age [11]. Since 1983, there has been much advancement in pre, intra, and postoperative care in HLHS. With progress in the field of cardiac surgery and introduction of various modifications in the stage treatment, there is a significant improvement in survival rates [12]. Despite improving results and more surgical procedures, children with HLHS require constant multi-specialty adapting care.

## Norwood procedure

The aim of this procedure is to use the right ventricle as the main pumping ventricle for systemic circulation. To achieve this goal, the following steps in the operation are taken:

i. Connection between the right ventricle and aorta is created with the aid of a pulmonary homograft (neoaorta).
ii. Developing a new source of pulmonary blood flow via the systemic-to-pulmonary artery shunt: modifed Blalock-Taussig (MBT) shunt or right ventricle-to-pulmonary artery (RV-PA) shunt. The shunt is a long-term replacement for prostaglandin E1 (PGE1), which was used preoperatively for PGE1-dependent ductus arteriosus (DA) patency.
iii. An atrial septectomy to secure good atrial communication, which is required to permit pulmonary venous return to reach the right ventricle. If restrictive atrial septum is present, then the Rashkind procedure along with stent implantation should be performed.

At conclusion, the right ventricle provides blood to both circulation systems in parallel, as opposed to two-ventricle physiology where blood flow is in-series. Ideally, blow flow should have a similar ratio in both circulation systems, i.e., Qp:Qs at 1:1.

In the Norwood anatomy and physiology, there is mixing of blood due to atrium septum. As a result, the typical arterial oxygen saturation values in these children are in the range of 75–85%. Such lower arterial oxygen saturation values (as compared with normal individuals) are surprisingly well-tolerated.

The source of pulmonary blood flow (PBF) by using a Gore-Tex(polytetrafluorethylene) tube implanted between the right innominate or subclavian artery and the right pulmonary artery constitute modifications of the Blalock-Taussig (B-T) shunt. The modification is based on a description by Alfred Blalock and Helen Taussig in 1945 [13, 14]. This modified shunt is known as the MBT shunt. The shunt is typically 3.5 mm in diameter, so that there is optimal flow and excessive flow to the lung is avoided. The MBT shunt allows for blood flow also in diastolic phase into the pulmonary vasculature and away from the coronary perfusion, which gives rise to development of the coronary steal phenomenon. As a result, there is volume load on the single ventricle and a decreased myocardial function, arrhythmias, and sudden cardiac death [15]. Acute shunt thrombosis—partial or total—is also a possible complication which may generate life-threatening hypoxemia [16]. To regulate these detrimental physiological features, as an alternative to the MBT shunt, the RV-PA shunt was developed.

Anastomosis between the right ventricle and pulmonary artery trunk is known as the RV-PA shunt, located on the right or left side of reconstruction of the neoaorta. Norwood and colleagues initially described the RV-PA conduit, but the reported method was long ignored [17]. Earlier, there had been many attempts at creating a RV-PA shunt in single ventricle patients [18–20].The RV-PA conduit has seen a dramatic revival in popularity since its re-introduction by Sano [21]. RV-PAs have an advantage as compared to MBT shunt because it prevents coronary steal during diastole that could potentially contribute to the improvement of treatment outcomes in the inter-stage period [22, 23].The RV-PA shunt requires a right ventriculotomy and the non-valved nature of the conduit may lead to regurgitant flow, which could potentially lead to ventricular dysfunction in an already stressed univentricular heart. These disadvantages may cause ventricular arrhythmias and impair the systemic right ventricle function [24]. The choice between these two sources of PBF is currently surgeon or institution-dependent.

Many single institutions published articles, comparing the traditional B-T shunt vs. the RV-to-PA conduit [25, 26]. Because of the conflicting results and limitations due to single-center retrospective studies, trials were needed for solving this question. The Pediatric Heart Network published a multicenter randomized trial in infants with HLHS undergoing the Norwood procedure, known as the Single Ventricle Reconstruction (SVR) trial [27]. In this trial, 555 patients were randomized to either a MBT shunt or an RV-PA conduit groups. There were 72 deaths or cardiac transplants in the RV-PA shunt group, and 100 deaths or cardiac transplants in the MBT shunt group. On the basis of the data, the RV-PA shunt as compared to the MBT was found to be associated with an improved transplantation-free survival at 12 months.

Two studies from SVR trials described in-hospital and inter-stage mortality associated with the Norwood procedure as being 16% irrespectively of shunt type. The inter-stage mortality between stage I and II was 6% for the RV-PA conduit, and 18% for the MBT shunt. The 3-year follow-up for transplant-free survival in the SVR trial did not differ by shunt types [28].

The most recent study identified an association between digoxin and reduced mortality following hospital discharge after the Norwood procedure using SVR data [29].

A study that investigated preoperative risk factors, using the database from the Society of Thoracic Surgeons, found that weight below 2.5 kg, preoperative shock, non-cardiac or genetic abnormalities, preoperative mechanical ventilatory or circulatory support, a small ascending aorta, an intact/restrictive interatrial septum, and the variant of HLHS with aortic atresia and mitral stenosis led to postoperative complications [30].

In a recent study from the Congenital Heart Surgeon’s Society, the propensity scores were used to compare 169 RV-PA conduit with 169 MBT shunt patients. There was better 6-year survival with the RV-PA (70%) conduit versus the MBT shunt (55%). The MBT shunt showed a lower transplant-free survival and right ventricular dysfunction with moderate to severe atrioventricular valvular regurgitation [31].

## Hybrid procedures

The hybrid procedure represents a less intensive approach that combines interventional cardiac catheterization with off-cardiopulmonary bypass surgery, first reported in 1993 [32]. Cardiopulmonary bypass use in neonates with cardiac and non-cardiac comorbidities might lead to neurological complications; thus, an alternative to CBP became the origin of hybrid approach in HLHS patients.

The hybrid procedure achieves the same goals as the standard Norwood procedure in a less intensive manner, which can be described as follows:

i. systemic perfusion is maintained with a ductal arteriosus stent.
ii. unrestricted pulmonary venous return is accomplished with either surgical atrial septectomy or a balloon atrial septostomy.
iii. controlled (diminished) PBF is accomplished with pulmonary artery bands on the right and the left pulmonary artery.

After the inter-stage period, the comprehensive stage I + II includes the removal of the PA bands, removal of the ductal stent, and repair of the aortic arch and pulmonary arteries. Removing the ductal stent is probably the most challenging element. It takes a technique similar to an endarterectomy to safely extract the stent without injuring the descending thoracic aorta. Initially, the hybrid approach was used for high-risk infants. With formal or relative contraindications to the Norwood stage I operation, single-center studies showed the feasibility of the approach, and others started using it on standard risk patients as an alternative to the Norwood stage I procedure [33].

The pioneering work by Galantowicz and colleagues [34] was very illustrative in demonstrating what can be accomplished with the hybrid approach for HLHS. Sixty-two patients underwent the hybrid stage I procedure. The results were impressive, with the hospital survival after the hybrid stage I reaching 97.5%. In the inter-stage I–II interval, two patients died. The hospital survival during the comprehensive stage I + II was 92%. The most important point derived from this early experience is that in certain patients with unfavorable anatomy, namely those with aortic atresia and no antegrade flow to the coronary arteries, jailing by the ductal stent may create stenosis of the retrograde orifice to the transverse arch. These patients should be identified before intervention, because a ductal stent can acutely lead to head vessel and/or coronary artery flow compromise, potentially leading to death from myocardial infarction and circulatory collapse. The options are to offer these patients the standard Norwood procedure or to stent the retrograde orifice at the time of the patent ductus arteriosus (PDA) stent [35].

The group in Giessen recently described 182 babies that underwent this hybrid strategy [36]. At 10 years, the probability of survival is 77.8%. Aortic arch re-interventions were only needed in 16.7% of patients. A benefit of this approach was that several of the patients were able to be transitioned to the biventricular repair after the hybrid approach instead of the single ventricle palliation.

## Hybrid-bridge-to-Norwood

The need for more catheterizations and the concerns about removing the stent during the comprehensive stage I + II led to the revival of another sequence of operations once described by Dr. Norwood, called the hybrid-bridge-to-Norwood or the “salvage-bridge-to-Norwood” [37]. In high-risk HLHS neonates with concomitant cardiac and non-cardiac comorbidities, in whom the initial Norwood stage I operation is deemed prohibitive, the sequence starts with bilateral pulmonary artery bands in the early period after birth. The ductus is kept open with prostaglandins, instead of a mechanical stent. The Norwood stage I is then performed after the baby is stabilized, typically at about 2 weeks of age. The “hybrid-bridge-to-Norwood” or “salvage-to-Norwood” approach gives the baby time to recover and allows time for the surgeon to undertake the Norwood stage I at a more advantageous moment. Although the in-hospital mortality with the “salvage-to-Norwood” approach is high as compared to other approaches in neonates with HLHS, it is the only alternative to certain death in an otherwise very high-risk and unstable situation.

## Inter-stage period

The period of time between hospital discharge after the Norwood procedure and the stage II surgery is commonly referred to as the inter-stage period. During the inter-stage period, accurate monitoring of the patient and cardiologic examination is important. After implementing parental education and home monitoring program (measuring systemic blood oxygenation with pulse oximeter, and changes in weight), there is a reduced complication rate and better survival [38]. Mortality between stages is 4–15%. The main causes of death are as follows: ventricular dysfunction, stenosis of the carotid artery or aortic arch, restriction of the atrial septum, coronary circulation disorders, and stenosis or systemic-pulmonary systemic obstruction [39].

The most common re-interventions after the first stage of treatment are also associated with problems. Stenosis of the aorta appears with an incidence of about 5–10% and in the majority of cases, it is effectively resolved by the method of transcutaneous balloon plastic surgery. Invasive cardiology techniques are also used for implantation of stents into the formation of stenosis of the anastomosis. Tricuspid valve surgery and interventions within pulmonary stenosis are performed during the second stage of treatment [35].

## Second stage of treatment

Currently, two basic surgical techniques are used: the hemi-Fontan operation or bidirectional Glenn anastomosis. The energy loss in the lateral intra-atrial tunnel (the third stage of treatment) produced after the previous hemi-Fontan operation is significantly lower compared to the energy dissipation in the tunnel produced after Glenn’s two-way operation. More physiological distribution of blood to both lungs after the hemi-Fontan surgery has been also noted [40]. The second stage of treatment after previous treatment hybridization is a more complicated and longer operation. It is a combination of the Norwood operation and hemi-Fontan operation and in some centers also serves as preparation for performing the third stage of invasive cardiological defect treatment [34].

The Norwood procedure is undertaken during the time when the pulmonary vascular resistance is too elevated to allow a cavopulmonary anastomosis. The second stage is usually undertaken between 4 and 6 months of age. The goal of the second stage is to unload the right ventricle. This is accomplished with either the bidirectional Glenn or the hemi-Fontan. The advantage of the bidirectional Glenn is that it can potentially be performed off-pump and is an easier connection. The hemi-Fontan makes the final stage more straightforward. This second stage originally was proposed as an interim palliation only for high-risk babies before undergoing the Fontan operation [41], instead of proceeding directly from a shunted physiology to total cavopulmonary connection **(**TCPC) in one step, which is a huge physiological change associated with a high risk of failure. After the staged palliation with the interim Glenn operation, breaking the adaptation to new cardiopulmonary flows into two lower-risk steps with better results, the Glenn operation became a standard staging procedure even in babies with a low-risk profile, leading to the current three-stage approach. The goal is to unload the ventricle as early as possible, minimize the potential steal from coronary blood flow, and limit the amount of time the pulmonary vasculature is exposed to systemic pressures before the baby can tolerate the Fontan [42].

Traditionally, the bidirectional Glenn anastomosis was situated between the superior vena cava and the pulmonary arteries. The inferior vena cava to pulmonary artery anastomosis was abandoned in the animal lab by Dr. Glenn after repeated failures in his animal models. In patients with unfavorable upper body systemic venous anatomy, the superior vena cava *(*SVC*)-*pulmonary artery (PA) connection is suboptimal or not feasible, and an alternative is needed to unload the heart. As it was demonstrated by studies, this subset of patients may benefit from the primary inferior vena cava (*I*VC)-pulmonary artery (PA) connection, the “Southern Glenn,” which we have performed successfully in two patients [43].

## Third stage of treatment—Fontan procedure

The Fontan operation typically connects the IVC to the right pulmonary artery (RPA) leading to a total cavopulmonary connection so that all PBF is achieved passively. In-series circulation is restored and saturation achieves near-normal levels. This typically happens between 24 and 48 months of age. There are numerous single institution series investigating the short- and long-term outcomes and predictors of mortality and morbidity after the Fontan completion [44, 45]. The consistent predictors of poor outcome across multiple studies are longer cross-clamp times, longer duration of hospital stay, heterotaxy, and atrioventricular valve anomaly.

The concept of the Fontan operation was first applied to a patient with tricuspid atresia by Francis Fontan in 1968 [46], but it has since become the final surgical stage for patients with HLHS. The goal of this ingenious operation is to direct the desaturated systemic venous return from the inferior vena cava into the pulmonary circulation, which may be done either via a lateral tunnel or extracardiac conduit that connects the inferior vena cava to the pulmonary artery. In some patients, small fenestration may be created in the Fontan conduit, a modification aimed at providing children with this unique anatomy and physiology with another means to fill their single ventricle, albeit with desaturated blood [47]. This practice may be especially helpful in patients with relatively high pulmonary vascular pressure values, although studies attempting to discern the merits of these theoretical benefits have been conflicting [47, 48].

Total venous-pulmonary connection may also be done by intervention cardiology via a polytetrafluorethylene-covered stent (covered stent) [49]. Balloon expansion under the control of angiography and echocardiography allows for an accurate placement of the stent in the correct position. This procedure requires prior preparation in II stage of treatment. The advantage is it may be done without ECMO support.

The strategy of using hybrid procedures will apply to few subsets and will not be a norm. Moreover, majority of centers are not very comfortable with Norwood Glen at stage 2 with retrieval of the stent to repair the arch which seems possible but likely to have sequels as arch obstruction and pulmonary artery plasty following the bilateral PA bands, pulmonary artery anatomy being the most important factor for influencing the outcome of the single ventricle repair. The sequential surgical procedures with good outcomes practiced in a good number of centers in the world are presented in (Table 1).

**Table 1:** The sequential surgical procedures for single-ventricle palliation (SVP)

Study name	The sequential surgical procedures
Emani et al. 2012 [50]	Stage 1 - > BDG - > Fotan - > cardiac transplantation OR Stage 1 - > BDG - > biventricular conversion - > cardiac transplantation.
Malec et al. 2000 [51]	Stage 1 with mBTS - > stage II (hemi-Fontan) and stage III (modified Fontan).
McGuirk et al. 2006 [52]	Norwood procedure - > bidirectional Glenn procedure (stage II) - > a modified Fontan procedure (stage III)
Andrews et al. [53]	stage I (the Norwood operation) - > stage II (the hemi-Fontan operation - > stage III (the Fontan operation - > cardiac transplantation
Rogers et al. 2012 [54]	stage I (mBTS) - > stage II (BDG) - > stage III extracardiac conduit (ECC) Fontan - > cardiac transplantation or stage I (RV-PA)- > stage II (the hemi-Fontan operation - > stage III (the Fontan operation - > cardiac transplantation
Malec et al. 2017 [55]	stage I (mBTS) - > stage II (BDG) - > stage III extracardiac conduit (ECC) Fontan - > cardiac transplantation or stage I (RV-PA)- > stage II (the hemi-Fontan operation - > stage III (the Fontan operation - > cardiac transplantation
Rai et al. 2018.[56]	stage I (RV-PA)- > stage II (the hemi-Fontan operation - > stage III (the Fontan operation - > cardiac transplantation

*BDG* bidirectional Glenn procedure, *mBTS* modifed right Blalock-Taussig shunt, *ECC* extracardiac conduit, *IALT* intra-atrial lateral tunnels, *HF* hemi-Fontan, *RV-PA* the right ventricle-to-pulmonary artery shunt

An unsolvable problem remains in any path taken (Surgical/interventional/Hybrid), for the staged palliation which is a ventricular function which is the real Achilles heel of these repairs. The possibility of use of a ventricular pump in future might be a reality to sort out this issue.

## Conclusions

There are widespread modifications of the Norwood operations which significantly improve HLHS results. Early diagnostic management, prenatal interventions, innovative diagnostic methods, constantly modified surgical techniques, and hybridization contribute to a significant progress in treatment options. This will allow for defining an optimal strategy of improving survival and quality of life in HLHS patients. The world experience and a lot of the available literature say that. The 1st-stage procedure could be done now safely either directly or with a bridge to Norwood followed by the stage 2 with a Glen or Hemi-Fontan and followed by a Fontan down the lane surgically.
